# Portal Decompression Using the Inferior Mesenteric Vein

**DOI:** 10.1155/1998/39129

**Published:** 1998

**Authors:** Paolo Gorini, Kaj Johansen

**Affiliations:** 1 Departments of Surgery Ospedale Borselli Ferrara Italy; 2 University of Washington School of Medicine Seattle USA

## Abstract

We report five patients with variceal hemorrhage, in three cases secondary to diffuse thrombosis of the portal, superior mesenteric and splenic veins. Mesenteric angiography demonstrated patency of the inferior mesenteric vein (IMV) in each, and successful portal decompression by anastomosis of the IMV to the left renal vein (*n*=4) or the inferior vena cava (*n*=1) was accomplished. Bleeding was permanently controlled: four patients have survived from one to eight years post-operatively. Because shunt procedures utilizing the IMV are technically straightforward, subtotally decompress the portal system and avoid the right upper quadrant, they may be advantageous in certain clinical settings.


*HPB Surgery*, 1998, Vol. 10, pp. 365–370
Reprints available directly from the publisher
Photocopying permitted by license only

© 1998 OPA (Overseas Publishers Association)
Amsterdam B.V. Published under license
under the Harwood Academic Publishers imprint,
part of The Gordon and Breach Publishing Group.
Printed in India.

### Portal Decompression Using the Inferior Mesenteric Vein

PAOLO GORINI and KAJ JOHANSEN

*Departments of Surgery, Ospedale Borselli, Ferrara, Italy, and University of Washington School of Medicine, Seattle, USA*

*(Received 1 August 1996; In final form 10 February 1997)*

**We report five patients with variceal hemorrhage, in three cases secondary to diffuse thrombosis of the portal, superior mesenteric and splenic veins. Mesenteric angiography demonstrated patency of the inferior mesenteric vein (IMV) in each, and successful portal decompression by anastomosis of the IMV to the left renal vein (*n*=4) or the inferior vena cava (*n*=1) was accomplished. Bleeding was permanently controlled: four patients have survived from one to eight years post-operatively. Because shunt procedures utilizing the IMV are technically straightforward, subtotally decompress the portal system and avoid the right upper quadrant, they may be advantageous in certain clinical settings.**

*Keywords:* Mesenteric venous thrombosis, portal hypertension, portasystemic shunt, inferior mesenteric vein

### INTRODUCTION

No single operative treatment is optimal for variceal hemorrhage arising from portal hypertension. Standard portacaval shunts reliably control recurrent variceal hemorrhage, but may be accompanied by an unacceptably high risk of post-shunt encephalopathy [1,2]. Further, a later attempt at liver transplantation may be complicated by the adhesions and anatomic distortion resulting from the prior right upper quadrant operation [3–5]. Selective shunts avoid the hilum of the liver but have a higher risk of variceal rebleeding [6] and may result in portal vein thrombosis [7]. Occasionally, diffuse splanchnic venous thrombosis [8,9] thwarts consideration of any conventional shunting procedure.

In several such clinical settings we have been successful in utilizing the inferior mesenteric vein (IMV) as a portal decompressive conduit. The operative procedure is technically straightforward, appears to provide reliable reduction of portal pressure, avoids post-shunt neurologic complications, and could be advantageous in patients who may later be candidates for liver transplantation.

### CASE REPORTS

Patient 1. A thirty-four year old woman with massive recurrent upper gastrointestinal hemor-

Correspondence: Providence Medical Center and The Hope Heart Institute, P O Box 34008, Seattle, USA 98124 Telephone (206) 320-2281 Fax (206) 320-3589.

rhage, endoscopically seen to arise from gastric varices, was referred for definitive care. She had biopsy-proven post-necrotic cirrhosis. Splanchnic angiography demonstrated a large IMV. Because of the patient's youth she was deemed to be a future liver transplant candidate, so a shunt procedure which avoided the right upper quadrant was felt warranted.

Exploration confirmed a patent IMV which accepted a 7 mm probe. The IMV was anastomosed side-to-side to the ventral surface of the left renal vein, after which a strong thrill could be palpated in the inferior wall of the renal vein. A pressure gradient of 8 mm Hg was measured between the splenic vein and the left renal vein. Post-operative angiography and duplex sonography [10] confirmed shunt patency, and the patient remains well, without evidence for encephalopathy or recurrent gastrointestinal hemorrhage, at 4 years follow-up.

Patient 2. A forty-six year old man was referred because of variceal hemorrhage. At age 24 the onset of massive upper gastrointestinal hemorrhage had led to the diagnosis of variceal bleeding secondary to diffuse splanchnic venous thrombosis, and he underwent resection of the gastric cardia with interposition of an isoperistaltic segment of jejunum [11]. He had no further evidence for gastrointestinal hemorrhage or liver disease for twenty-two years. He then suffered several episodes of hematemesis and upper endoscopy demonstrated large esophageal and gastric varices. Splanchnic angiography confirmed the diagnosis of diffuse thrombosis of the portal, superior mesenteric, and splenic veins, but he was noted to have a moderate-sized (5 mm) IMV. Abdominal exploration confirmed a patent IMV: this was anastomosed to the left renal vein using an interposed saphenous vein graft. Endoscopic examination one week and four years post-operatively demonstrated 1+ esophageal varices and no gastric varices; he has had no further upper gastrointestinal hemorrhage at 12 years follow-up.

Patient 3. A 39-year old man had massive recurrent bleeding from esophageal and gastric varices due to alcoholic cirrhosis. Considered a potential liver transplantation candidate, he required urgent portal decompression. Splanchnic angiography demonstrated a 6 mm IMV; he underwent an uneventful IMV-left renal vein anastomosis with satisfactory portal decompression (corrected splenic collateral vein pressure 12 mm Hg) and no further bleeding. However, post-operatively he developed fulminant hepatic failure and expired on the 20th post-operative day while awaiting liver transplantation.

Patient 4. A 56-year old man had undergone an unknown procedure – probably a mesocaval shunt – 15 years previously to treat bleeding esophageal varices. Abstinent from alcohol for more than a decade, he presented with variceal hemorrhage which recurred despite several sessions of endoscopic sclerotherapy. Splanchnic angiography revealed portal and superior mesenteric vein thrombosis, a patent splenic vein, and a large IMV. Side-to-side anastomosis of the IMV to the inferior vena cava was performed without complication. No further bleeding occurred, and upper endoscopy at one and three years showed stable 1+ esophageal varices.

Patient 5. A 55-year old man was referred following massive rectal bleeding, seen on sigmoidoscopy to be arising from hemorrhoidal varices. Splanchnic angiography demonstrated an occluded portal, splenic, and superior mesenteric vein but a large and patent IMV. Coagulation workup revealed anti-thrombin III deficiency [9]. A side-to-side IMV-left renal vein anastomosis was performed. Duplex scan at 3 days and 6 months demonstrated a patent inferior mesorenal shunt. No further rectal bleeding has occurred at 5 years follow-up: the patient has been maintained on warfarin to treat his prothrombotic state.

### COMMENT

Optimal surgical therapy for variceal hemorrhage depends on the procedure's urgency, the possibility of future liver transplantation, and the patency of the various splanchnic veins. Standard portacaval shunt procedures are highly effective at halting variceal hemorrhage, but have a substantial risk [30–80% [1,2]] of post-shunt neurologic complications, and in addition result in scarring and anatomic distortion in the right upper quadrant – a handicap if a subsequent liver transplantation is contemplated [3–5]. Various selective shunts – most notably the distal splenorenal shunt [12] – confer reasonable protection against further variceal bleeding, but are encumbered with numerous clinical contraindications and ever more complex technical considerations [13]. At least 10% of such patients will suffer portal vein thrombosis [7], thereby markedly complicating subsequent liver transplantation. Further, when compared to standard portacaval shunts in properly-designed prospective randomized trials [14], selective shunts do not provide definitive protection against post-shunt neurologic complications, especially in that majority of Western patients who are alcoholics [15].

Other operative procedures to prevent variceal bleeding, most notably various forms of esophagogastric devascularization as popularized by Sugiura [16], have had excellent results in Japan, but these have not been confirmed either in Western Europe or in North America.

Use of IMV for portal decompression has been reported sporadically in the past [17–20] – most commonly in patients is whom diffuse splanchnic venous thrombosis [8,9] interdicted standard shunt procedures, or in patients in whom prior splenectomy prevented the performance of distal splenorenal shunt [20]. These indications remain relevant, but we have resurrected the IMV shunt because of two newer additional considerations – development of the concept of *partial portal decompression* and the maturation of *liver transplantation* as a clinically feasible management of patients with liver disease and variceal hemorrhage.

A body of recent experimental [21] and clinical [22–24] data suggest that, in comparison to total shunts, procedures which only partially decompress the portal system may result in a lesser risk of post-shunt liver failure and neurologic deterioration. Some investigators suggest that this beneficial effect results from continued prograde portal flow [24], consonant with the "selectivity" principle originally characterized by Warren [12]. Other studies, however, suggest that the protective effect of partial portal decompression is due to diminished intestinal neurotoxin absorption in the presence of residual mesenteric venous hypertension [21,22]. Regardless, shunts which only partially decompress the portal system appear to result in a lower risk of post-shunt morbidity and mortality [22–24]. This principle has been applied to the concept of transjugular intrahepatic portacaval shunting (TIPS), a nonsurgical transcatheter technique in which a 10 mm Hg post-shunt gradient between portal vein and supra-hepatic inferior vena cava is considered optimal [26].

The increasing availibility of liver transplantation to treat end-stage liver disease means that certain variceal bleeders treated by shunt operations may later be transplant candidates [3]. While prior performance of a standard portacaval shunt is no longer an absolute contraindication to liver transplantation [25], the scarring and anatomic distortion from such procedures undoubtedly complicate the performance of subsequent liver replacement [3–5]. Consequently, the recommendation has been made that any such patient who might later require liver transplantation should undergo either mesocaval [27] or distal splenorenal shunts [4,5]. Because the former procedure has a substantial rate of graft thrombosis and recurrent variceal bleeding [28], and the latter procedure is technically taxing [13] and has multiple contraindications [29], an alternative shunt option would be very helpful. In certain patients TIPS may be an option: however, this procedure is not durable and may have substantial complications [30,31], sometimes necessitating operative portal decompression [31]. We view the IMV shunt as a rational alternative in such patients.

An important feature favoring the use of the IMV for portal decompression is its technical ease. Because of the proximity of the IMV and the left renal vein the IMV shunt may be the most technically straightforward of all portal decompressive procedures. The IMV dilates in portal hypertension [17], frequently to 5–6 mm in diameter and even more in certain conditions of diffuse splanchnic venous thrombosis where it may remain the only patent mesenteric outflow vessel [17–19]. Second, the procedure is performed entirely on the left side of the mesentery, leaving the right upper quadrant inviolate–useful if future orthotopic liver transplantation is contemplated. Third, because it is an exemplary "small-stoma" shunt, it only partially decompresses the portal system and should confer maximal protection against post-shunt encephalopathy [21–24]. Finally (particularly if the procedure is performed as an inferior mesocaval anastomosis, as in our Patient #4) excellent angiographic access to the coronary veins for subsequent embolization may be possible [32].

While longer follow-up of a larger group of patients is required to discern whether use of the IMV as a portal decompressive conduit provides longterm protection against variceal rebleeding **and** post-shunt neurologic complications, our experience suggests that it is a technically straightforward procedure with good short-term function. It may offer therapeutic advantages in selected patients with variceal hemorrhage who require portal decompression.

### *References*

[1] Langer, B., Taylor, B. R., Mackenzie, D. R. *et al.* (1985). Further report of a prospective randomized trial comparing distal splenorenal shunt with end-to-side portacaval shunt. *Gastroenterology*, **88**, 424–429.

[2] Reynolds, T. B., Donovan, A. J., Mikkelsen, W. P. *et al.* (1981). Results of a 12-year randomized trial of portacaval shunt in patients with alcoholic liver disease and bleeding varices. *Gastroenterology*, **80**, 1005–1011.

[3] Iwatsuki, S., Starzl, T. E., Todo, S. *et al.* (1988). Liver transplantation in the treatment of bleeding esophageal varices. *Surgery*, **104**, 697–705.

[4] Brems, J. J., Hiatt, J. R., Klein, A. S. *et al.* (1989). Effect of a prior portasystemic shunt on subsequent liver transplantation. *Ann. surg.*, **209**, 51–56.

[5] Mazzaferro, V., Todo, S., Tzakis, A. *et al.* (1990). Liver transplantation in patients with previous portasystemic shunt. *Am. J. Surg.*, **160**, 111–116.

[6] Eckhauser, F. E., Pomerantz, R. A., Knol, J. A. *et al.* (1986). Early variceal rebleeding after successful distal splenorenal shunt. *Arch. Surg.*, **121**, 547–552.

[7] Jin, G. and Rikkers, L. E. (1991). The significance of portal vein thrombosis after distal splenorenal shunt. *Arch. Surg.*, **126**, 1011–1016.

[8] Gertsch, P., Mathews, J., Lerut, J. *et al.* (1993). Acute thrombosis of the splanchnic veins. *Arch. Surg.*, **128**, 341–345.

[9] Karl, R., Garuck, I., Zarms, C. *et al.* (1981). Surgical implications of antithrombin-III deficiency. *Surgery*, **89**, 429–433.

[10] Helton, W. S., Montana, M., Dwyer, D. and Johansen, K. H. (1988). Duplex sonography accurately assesses portacaval shunt patency. *J. Vasc. Surg.*, **8**, 657–660.

[11] Merendino, K. A. and Dillard, D. H. (1955). The concept of sphincter substitution by an interposed jejunal segment for anatomic and physiologic abnormalities at the esophagogastric junction, with special reference to reflux esophagitis, cardiospasm, and esophageal varices. *Ann. Surg.*, **142**, 486–492.

[12] Warren, W. D., Zeppa, R. and Fomon, J. J. (1967). Selective transplenic decompression of gastroesophageal varices by distal splenorenal shunt. *Ant. Surg.*, **166**, 437–455.

[13] Warren, W. D., Millikan, W. J., Henderson, J. M. *et al.* (1986). Splenopancreatic disconnection. Improved selectivity of distal splenorenal shunt. *Ann. Surg.*, **204**, 346–355.

[14] Grace, N. D., Conn, H. O., Resnick, R. H. *et al.* (1988). Distal splenorenal vs. portal-systemic shunts after hemorrhage from varices: a randomized controlled trial. *Hepatology*, **8**, 1475–1488.

[15] Zeppa, R., Hensley, G. T., Levi, J. U. *et al.* (1978). The comparative survival of alcoholics versus non-alcoholics after distal splenorenal shunt. *Ann. Surg.*, 187–510.

[16] Sugiura, M. and Futagawa, S. (1984). Esophagogastric transection with paraesophagogastric devascularization in the treatment of esophageal varices. *World. J. Surg.*, **8**, 673–676.

[17] Mozes, M., Tzur, N. and Bogokowsky, H. (1967). Mesenterorenal shunt for decompression of portal hypertension. *Surgery*, **62**, 884–887.

[18] Drews, J. A. and Castagna, J. (1976). Inferior mesorenal shunt as a second procedure for portal decompression. *Surg. Gynecol. Obstet*, **142**, 84–86.
[19] Alvarez, F., Bernard, O., Brunelle, F. *et al.* (1983). Portal obstruction in children. II. Results of surgical portosystemic shunts. *J. Pediatr.*, **103**, 703–707.
[20] Warren, W. D., Millikan, W. J., Henderson, J. M. *et al.* (1984). Selective variceal decompression after splenectomy or splenic vein thrombosis. *Ann. Surg.*, **199**, 696–702.
[21] Johansen, K. H., Girod, C., Lee, S. S. and Lebrec, D. (1990). Mesenteric venous stenosis reduces hyperammonemia in the portacaval-shunted rat. *Eur. Surg. Res.*, **22**, 170–174.
[22] Johansen, K. (1992). Prospective comparison of partial versus total portal decompression for bleeding esophageal varices. *Surg. Gynecol. Obstet*, **175**, 528–534.
[23] Rikkers, L. F. (1983). Portal hemodynamics, intestinal absorption and post-shunt encephalopathy. *Surgery*, **94**, 126–133.
[24] Rosemurgy, A. S., Goode, S. E. and Camps, M. (1996). The effect of small-diameter H-graft portacaval shunts on portal blood flow. *Am. J. Surg.*, **171**, 154–156.
[25] AbouJaoude, M. M., Grant, D. R., Ghent, C. N. *et al.* (1991). Effect of portasystemic shunts on subsequent transplantation of the liver. *Surg. Gynecol. Obstet*, **172**, 215–219.
[26] Kerlan, R. K., LaBerge, J. M., Gordon, R. L. and Ring, E. J. (1995). Transjugular intrahepatic portosystemic shunts: current status. *AJR.*, **164**, 1059–1066.
[27] Smith, R. B., Warren, W. D., Salam, A. A. *et al.* (1980). Dacron interposition shunts for portal hypertension: an analysis of morbidity correlates. *Ann. Surg.*, **192**, 9–17.
[28] Millikan, W. J., Warren, W. D., Henderson, J. M. *et al.* (1985). The Emory prospective randomized trial: selective versus non-selective shunt to control variceal bleeding. Ten year follow-up. *Ann. Surg.*, **201**, 712.
[29] Henderson, J. M., Millikan, W. J. and Galloway, J. R. (1990). The Emory perspective of the distal splenorenal shunt in 1990. *Am. J. Surg.*, **160**, 54–59.
[30] Helton, W. S., Belshaw, A., Althaus, S. *et al.* (1993). Critical appraisal of the angiographic portacaval shunt (TIPS) *Am. J. Surg.*, **165**, 566–571.
[31] Lauden, S. and Launois, B. (1995). Derivations portocaves de sauvetage après echec de shunt intrahepatique. *Ann. Chir.*, **49**, 324–326.
[32] Coldwell, D. M., Moore, A. D. A., Ben-Menachem, Y. and Johansen, K. H. (1991). Bleeding gastroesophageal varices: coronary vein embolization following partial portal decompression. *Radiology*, **178**, 249–251.

### COMMENTARY

The authors resurrect an interesting but technically simple portosystemic shunt, namely, the inferior mesenteric vein to renal vein or vena caval shunt. The authors draw attention to reports from the 1960's and 1970's about the use of this shunt. They have used it in 5 patients with moderately long-term success in 4. They advocate this shunt for patients with diffuse splanchnic venous thrombosis and also for patients who are potential liver transplant candidates.

It needs to be recognized that the role of the inferior mesenteric vein shunt is as yet unproven, despite earlier reports and the present paper. However, it is a useful alternative to be kept in mind for a difficult patient situation. I do not believe that this shunt is justified in patients who are likely to be transplant candidates and who have an otherwise suitable portal venous system for a standard distal splenorenal shunt or a Sarfeh narrow-diameter PTFE shunt (this latter being placed from the mesenteric vein to the vena cava rather than in the standard portacaval position). However, in patients with diffuse splanchnic venous thrombosis, if the inferior mesenteric vein is patent, it seems a reasonable alternative in endoscopic failures. The authors have also used the procedure in one patient with uncontrollable rectal haemorrhage from anorectal varices. This could also be an important indication.

Our group's current view is that endoscopic therapy is the primary treatment of choice for acute bleeding varices. We agree with the shift that is occurring to variceal banding or a combination of banding and sclerotherapy from the previously widely used pure sclerotherapy techniques [1]. We define failures as patients who have two further bleeds after endoscopic management during a single hospital admission for acute variceal bleeding. We currently submit such patients to an emergency TIPS procedure. It should be noted that this is rarely undertaken, because the percentage of patients failing endoscopic therapy in our institution is currently very low [2]. If a patient with diffuse splanchnic venous thrombosis, including portal vein thrombosis was to fail endoscopic therapy, then the authors' suggestion of a surgical inferior mesenteric vein shunt, if this vein is patent and enlarged, seems reasonable.

We currently advocate either the distal splenorenal shunt or the narrow diameter PTFE shunt of Sarfeh [3] for the failures of endoscopic therapy in long-term management after a variceal bleed.

### *References*

[1] Terblanche, J., Stiegmann, G. V., Krige, J. E. J. and Bornman, P. C. (1994). Long-term management of variceal bleeding: The place of varix injection and ligation. *World Journal of Surgey*, **18**, 185–192.

[2] Bornman, P. C., Krige, J. E. J. and Terblanche, J. (1994). Management of oesophageal varices. *Lancet*, **343**, 1079–1084.

[3] Sarfeh, I. J., Rypins, E. B. (1994). Partial versus total portacaval shunt in alcoholic cirrhosis. Results of a prospective, randomized clinical trial. *Annals of Surgery*, **219**, 353–361.

John Terblanche
Professor and Chairman
Department of Surgery
University of Cape Town and
Groote Schuur Hospital Teaching Group
Observatory 7925
Cape Town, South Africa

